# The Partner Perspective on Autologous and Implant-Based Breast Reconstruction

**DOI:** 10.1007/s00266-023-03286-2

**Published:** 2023-02-23

**Authors:** Maxi von Glinski, Nikla Holler, Sherko Kümmel, Christoph Wallner, Johannes Maximilian Wagner, Alexander Sogorski, Felix Reinkemeier, Mattea Reinisch, Marcus Lehnhardt, Björn Behr

**Affiliations:** 1 Department of Plastic and Hand Surgery, Burn Center, BG-University Hospital Bergmannsheil, Ruhr University Bochum, Buerkle-de-la-Camp Platz 1, 44789 Bochum, Germany; 2 Clinic for Gynecology with Breast Center, Charité-Universitätsmedizin Berlin, Luisenstraße 65, 10117 Berlin, Germany; 3 Breast Unit, Kliniken Essen-Mitte, Henricistraße 92, 45136 Essen, Germany

**Keywords:** Breast cancer, Breast reconstruction, Partner satisfaction with breast reconstruction, Autologous breast reconstruction, Implant-based breast reconstruction, Patient-reported outcomes

## Abstract

**Introduction:**

Partner involvement in the decision-making process concerning breast reconstruction (BR) after a breast cancer diagnosis may be very supportive for the patient. So far, no study evaluates partner satisfaction with the outcome after BR and the relationship to patient satisfaction. The aim of this study was to assess and compare partner satisfaction of BR with autologous tissue (ABR) and prosthetic implants (IBR), respectively, and compare it to patient-reported outcomes.

**Patients and Methods:**

All patients undergoing ABR and IBR between January 2014 and December 2020 were asked to participate with their partners. Patient and partner satisfaction with breast reconstruction, overall outcome as well as patient’s perceived and self-reported psychosocial well-being were evaluated using the Breast-*Q* and a modified partner questionnaire, respectively.

**Results:**

Fifty-three couples participated (IBR: *n*=30, ABR: *n* = 23). Patient and partner satisfaction with breast (*r* = 0.552), outcome (*r* = 0.465) as well as patient’s perceived and self-report psychosocial well-being (*r* = 0.495) were highly correlated with partners scoring significantly higher (*p*<0.001). In terms of partner satisfaction, both reconstructive procedures achieved satisfactory results. ABR scored higher in terms of softness of breast and how natural the breast feels to touch whereas IBR was rated superior evaluating the breast size.

**Conclusion:**

Both reconstructive procedures achieve satisfactory results in terms partner satisfaction whereas patient’s psychosocial well-being was highly overestimated by their partners. Hence, partner inclusion in the regular psycho-oncological support might further sensitize them of the high psychological burden of a breast cancer diagnosis and therefore stabilize patients private support system.

**Level of Evidence III:**

This journal requires that authors assign a level of evidence to each article. For a full description of these Evidence-Based Medicine ratings, please refer to the Table of Contents or the online Instructions to Authors www.springer.com/00266.

## Introduction

Despite continuous optimization of treatment options, a mastectomy is still indicated in approximately 30–45% of breast cancer patients [1, 2].

The most common approaches for breast reconstruction involve prosthetic implants (IBR) and free autologous tissue transfer (ABR) [3, 4]. IBR still presents the most common reconstruction procedure [3, 5] even though the literature suggests better patient-reported outcomes in ABR [6].

The breast is generally regarded as a symbolic expression of femineity, motherhood and attractiveness [7]. Consequently, a mastectomy does not only present a major intervention in patient’s physical well-being, but may lead to psychosexual problems and depression [8].

In the long-term, mastectomy may be accompanied by painful memories of the malignant disease, a changed perception of body image and thus a reduced self-esteem [9, 10].

Therefore, breast reconstruction is considered a fundamental part of breast cancer therapy, which is reimbursed by health insurance companies and serves to psychologically process the disease and improve quality of life (QoL) [11]. The Italian psychiatrist Wili Pasini, founder of the European Federation of sexology, described the breast reconstruction (BR) as a “psychotherapeutic measure” [12]. This statement emphasizes the constantly increasing number of patients undergoing breast reconstruction [2, 13].

Many women seem to have difficulties communicating their feelings about the mastectomy to their partners [14]. Poor communication can put a strain on partnerships in terms of unresolved conflicts and even the termination of the relationship [14]. It could be shown that support from partners helps patients to deal with the diagnosis [7, 15], which results in an improvement of their psychological well-being [9, 16] and accelerates recovery [16]. Whereas the importance of partner integration into the preoperative decision-making process has already been pointed out [17], studies investigating partner satisfaction with the outcome after BR and the effect on patient satisfaction are lacking [18]. The aim of this study is to evaluate partner satisfaction with the outcome after ABR and IBR, respectively, in context of the patient’s satisfaction with the outcome.

## Patients and Methods

Eligible patients had a time span since BR of at least one year before study inclusion, were free of cancer or metastases, were currently in a partnership and did not have a language barrier. Breast reconstruction comprised ABR or IBR between January 2014 and May 2020 following therapeutic or prophylactic mastectomy at the two participating study centers. Patients undergoing a mixed approach (e.g., bilateral reconstruction comprising unilateral IBR and unilateral ABR or combination with pedicled flap) were excluded.

Patients and their partners were contacted via mail and phone. After giving written informed consent patients completed the Breast-*Q*, their partners a modified questionnaire to evaluate their satisfaction with the outcome.

The Breast-*Q* (Reconstruction Module) is a widely used standardized questionnaire to assess patient-reported satisfaction with the outcome after BR [19]. We included the following domains: “Satisfaction with breast,” “Satisfaction with outcome” and “Psychosocial well-being.” Scores for each domain ranging from 0 to 100 with higher scores indicating greater satisfaction were generated with a survey-specific software.

The partner questionnaire comprised similar questions concerning their satisfaction with the reconstructed breast (4-point Likert scale: very dissatisfied, somewhat dissatisfied, somewhat satisfied, very satisfied), overall outcome (3-point Likert-scale: disagree, agree, strongly agree) as well as the perceived psychosocial well-being of the patients (5-point Likert scale: None of the time, a little of the time, some of the time, most of the time, all of the time). Furthermore, we evaluated satisfaction with the abdomen in partners of ABR patients (4-point Likert scale). To provide further comparison of partners satisfaction with the breast, outcome and psychosocial well-being of their female partners, we also generated sum scores for the beforementioned domains.

Patients clinical and surgical data were retrospectively obtained from the electronic medical chart. These included patients age, type of mastectomy and reconstruction, indication for mastectomy, cancer-related treatment such as radio- and chemotherapy and follow-up period. Partner-specific information such as partners age, whether the couple was married and had children were obtained via a telephone interview.

### Statistical Analysis

Data were analyzed using SPSS version 27 (IBM SPSS statistics, IBM Corporation, Armonk, New York/USA). Descriptive statistics and frequency distributions were generated for the sociodemographic, clinical and surgical characteristics of the sample size as well as partners satisfaction with the abdomen.

Independent *t* tests were used to evaluate differences in partner satisfaction with the breast, outcome and perceived patient’s psychosocial well-being depending on the method of breast reconstruction. The relationship of patient and partner satisfaction with the breast, outcome and patient’s perceived psychosocial well-being was measured using Pearson’s correlation coefficient and the *t* test for dependent variables, respectively.

A two-sided *p* value of <0.05 was considered statistically significant.

The structure and content of the manuscript adhere to the STROBE guidelines for cohort studies.

## Results

At the time of study inclusion, 111 patients were in a permanent relationship. A total of 99 patients consented to participate with their partners out of which 53 couples (mean age patients 49.8 ± 7.5; mean age partners 53.1 ± 7.9) returned the questionnaire and were included in the study (response rate 47.7%). All sociodemographic and clinical characteristics of the patients and their partners are summarized in Table. 1. Twenty-three patients underwent uni- (*n* = 18) or bilateral (*n* = 4) ABR, whereas 30 patients underwent uni- (*n* = 18) or bilateral (*n* = 12) IBR. All patients included had a history of therapeutic mastectomy with 26.4% of them having contralateral prophylactic mastectomy. 19.7% of all patients underwent PMRT and 32.1% chemotherapy. Mean time of follow-up was 2, 5 years (30.7 months ± 13.5).

**Table 1.** Sociodemographic and clinical characteristics of all patients undergoing primary breast reconstruction

	Patient, *N*=53
Procedures, *N* (%)	68
Prepectoral Implant	42 (61.8)
DIEP	25 (36.8)
Ms-TRAM	1 (1.5)
Mean age ± SD patients, yr (range)	49.8 ± 7.5
Mean age ± SD partner, yr (range)	53.1 ± 7.9
Partnership	
Married	46 (86.8)
Children	47 (88.7)
From current relationship	41 (77.4)
From previous relationships	6 (11.3)
Laterality, *N* (%)	
Unilateral	37 (69.8)
Bilateral	16 (30.2)
Indication, N (%)	
Prophylactic	0
Therapeutic	53 (100.0)
contralateral prophylactic mastectomy^b^	14 (26.4)
Type of mastectomy, *N* (%)	
Radical	5 (7.4)
Nipple-sparing	46 (67.6)
Skin-sparing	17 (25.0)
PMRT^c^, *N* (%)	
Before reconstruction	11 (15.5)
After reconstruction	3 (4.2)
History of chemotherapy, *N* (%)	17 (32.1)
Follow-up, month (mean, SD)	30.7 ± 13.5

*SD*: standard deviation; *p<0.05; **p<0.01

^b^counts of patients with a history of therapeutic mastectomy

^c^Postmastectomy radiation therapy

Table 2 summarizes the results of the partner questionnaire in detail.

**Table 2.** Partner questionnaire–autologous vs. implant-based breast reconstruction

Items (mean ± SD); Number of partner=62	ABR, *N*=23	IBR; *N*=30	*p* value
*Satisfaction with breast (4-point Likert scale)*			
a. How she looks in clothes? b. The shape of the reconstructed breast (s) when she wears a bra? c. How normal she looks in her clothes? **d. The size of her reconstructed breast?** e. With her able to wear clothing that is more fitted? f. How the breasts are lined up in relation to each other? g. How the reconstructed breast (s) looks like in comparison ± to the former natural breast? h. The softness of the reconstructed breasts? i. How equal in size the breasts are to each other? j. How natural the reconstructed breast (s) looks? k. How naturally the reconstructed breast (s) sits/hangs? **l. How the reconstructed breast (s) feels to touch?** **m. How much the reconstructed breast (s) feels like a natural part of her body?** n. How closely matched (similar) the breasts are to each other? o. How your partner looks unclothed? p. Sum score	3.65 ± 0.48 3.65 ± 0.48 4.00 ± 0.00 **3.35 ± 0.65** 3.52 ± 0.59 3.35 ± 0.65 3.35 ± 0.78 3.35 ± 0.65 3.30 ± 0.77 3.26 ± 0.62 3.33 ± 0.82 **3.35 ± 0.71** **3.35 ± 0.57** 3.13 ± 0.69 3.43 ± 0.51 71.7±15.6	3.85 ± 0.37 3.79 ± 0.41 3.90 ± 0.31 **3.72 ± 0.53** 3.71 ± 0.54 3.61 ± 0.57 3.31 ± 0.89 3.00 ± 0.83 3.28 ± 0.96 3.32 ± 0.77 3.34 ± 0.86 **2.86 ± 0.76** **2.82 ± 0.82** 3.25 ± 1.01 3.55 ± 0.74 73.1 ± 16.5	0.127 0.274 0.425 0.025* 0.229 0.134 0.874 0.348 0.908 0.762 0.976 0.022* 0.012* 0.619 0.519 0.761
*Satisfaction with abdomen (N=31) (4-point Likert scale)*			
a. How the abdomen of your partner looks when unclothed b. The position of the navel (belly button)? c. How the abdominal scars look? d. How the abdomen of your partner feels to touch in± comparison to the former abdomen before the surgery? e. How the abdomen of your partner looks in comparison to± the former abdomen before the surgery?	3.61 ± 0.72 3.96 ± 0.64 3.65 ± 0.71 3.65 ± 0.71 3.57 ± 0.73	- - - - -	- - - - -
*Satisfaction with outcome (3-point Likert scale)*			
a. A reconstructed breast is better than no breast? b. I would encourage other women in my partner´s situation to undergo breast reconstruction surgery. c. In this situation I would always again wish my partner for this breast reconstruction. d. I do not regret my partner had this breast reconstruction surgery. e. The breast reconstruction surgery has improved my partner´s quality of life. f. The result completely met my expectations. g. It turned out exactly as I expected. h. Sum score	2.87 ± 0.34 2.87 ± 0.34 2.91 ± 0.28 3.00 ± 0.00 2.70 ± 0.56 2.65 ± 0.57 2.50 ± 0.55 83.0±16.9	2.93 ± 0.25 2.87 ± 0.43 2.83 ± 0.46 2.83 ± 0.53 2.64 ± 0.62 2.39 ± 0.69 2.39 ± 0.69 79.5 ± 19.2	0.441 0.979 0.471 0.096 0.053 0.259 0.723 0.489
*Psychosocial well-being (5-point Likert scale)*			
a. Confident in a social setting? b. Emotionally able to do things that she wants to do? c. Emotionally healthy? d. Of equal worth of other women? e. Self-confident? f. Feminine in her clothes? g. Accepting of her body? h. Normal? i. Like other women? j. Attractive? k. Sum score	4.65 ± 0.57 4.74 ± 0.45 4.52 ± 0.67 4.52 ± 0.59 4.70 ± 0.47 4.55 ± 0.74 4.17 ± 0.72 4.57 ± 0.59 4.67 ± 0.52 4.22 ± 0.95 79.5 ± 14.9	4.67 ± 0.55 4.50 ± 0.94 4.23 ± 1.04 4.57 ± 0.84 4.50 ± 0.94 4.72 ± 0.53 4.13 ± 1.22 4.30 ± 1.18 4.45 ± 0.98 4.1 ± 1.25 80.1 ± 21.1	0.926 0.227 0.252 0.812 0.327 0.318 0.881 0.290 0.604 0.629 0.906

*SD*: standard deviation; *p<0.05; **p<0.01

Overall scores revealed partners to be satisfied with the reconstructed breast in almost all aspects evaluated (scores ≥3). Comparing the results of partners of ABR and IBR patients, respectively, it could be shown that IBR was rated superior in terms of breast size (*p* = 0.025), how the patient looks in clothes/more fitted clothes (n. s.) and how the breasts are lined up in relation to each other (*p* = 0.134). On the other hand, ABR scored significantly higher in terms of softness of the reconstructed breast (n. s.) breast haptic (*p* = 0.022) and how natural the reconstructed breast feels as part of the women’s body (*p* = 0.012). In contrast, within the IBR-group, breast haptic scored lower than 3 (somewhat dissatisfied–somewhat satisfied).

All other categories as well as the sum score revealed almost similar results in both cohorts.

The section ‘Satisfaction with Abdomen’ was only applicable in partners of ABR patients. Every subcategory scored higher than >3.5 which corresponds to a satisfactory result.

Ratings of ‘satisfaction with outcome’ revealed an overall satisfaction of partners of both patient cohorts with the decision to perform a BR. Partners of ABR patients were more likely to strongly agree with the statement that the breast reconstruction has improved their partner´s QoL (*p* = 0.053). Also, they tended to state more often that the result completely met their expectations (n. s.).

Average scores of all subcategories within the evaluation of the perceived psychosocial well-being of the patients were as high as 4-5 indicating that partners supposed that their female partners have felt most/all of the time confident/self-confident, emotionally stable and feminine. The lowest scores in this section were achieved in the subcategories ‘body acceptance’ (IBR: 4.13, ABR: 4.17) and ‘attractiveness’ (IBR: 4.1, ABR: 4.22). There were no relevant differences between the two patient cohorts.

To further compare the partner satisfaction with breast, outcome as well as the perceived psychosocial well-being of the patients (evaluated by their partners) with patients self-reported outcomes, we calculated sum scores for the beforementioned categories for both cohorts and performed Pearson Correlation Coefficient, Table. 3. Patient and partner scores of all three categories were highly correlated (*r*>0.46, *p*<0.001). At the same time, partners were significantly more satisfied with breast and the overall outcome compared to the patient herself (*p*<0.001). Furthermore, they significantly overestimated patient’s psychosocial well-being (*p*<0.001).

**Table 3.** Quality of life in patients after breast reconstruction and their partners

Items (mean ± SD)	Partners, *N*=53	Patients, *N*=53	Pearson correlation		*t* Test^b^ *p* value
			*r*^a^	*p* value	
Patient/partner satisfaction with breast outcome	72.5 ± 15.9 81.0 ± 18.2	64.02 ± 17.7 77.32 ± 18.7	0.552 0.465	<0.001** <0.001**	<0.001** <0.001**
Psychosocial well-being	79.8 ± 18.5	76.26 ± 20.4	0.495	<0.001**	<0.001**

*SD*: standard deviation; *p<0.05; **p<0.01; ^a^Pearson correlation coefficient; ^b^*t* test for dependent variables

## Discussion

A breast cancer diagnosis with subsequent mastectomy and BR presents a major psychological challenge for the patient [8]. It has been shown that patient’s self-image and psychological well-being are highly correlated with the support she experiences in her partnership [17, 20]. Sandham et. al did already underline the importance of partner integration into the preoperative decision-making process since the two most popular procedures—ABR and IBR—include various advantages and disadvantages which have to be weighed against each other [17]. But so far, partner satisfaction with the reconstructed breast depending on the procedure performed as well as the relationship with patient satisfaction has been neglected [18].

We could show that both procedures achieve good results in terms of partner satisfaction with breast, outcome, abdomen and the perceived psychological well-being of the patient. ABR scored higher in terms of breast softness and natural touch whereas IBR scored higher evaluating the size of the reconstructed breast(s). Patient and partner scores were highly correlated even though partners scored significantly higher in all subscales evaluated.

So far, only one study by Cimaroli et al. [18] did investigate partner satisfaction with the outcome after breast reconstruction as well as the relationship of partner and patient satisfaction with the outcome. They showed in a small study collective with just 11 couples included a high correlation of patient and partner satisfaction with the outcome administering the Breast-*Q* to the patients and a modified questionnaire to their partners. Even though they included both IBR and ABR patients, they did not compare self-report outcome parameter of both procedures [18].

Patient-reported outcomes in BR have widely been explored [5, 21]. Mean Breast-Q scores of our patient collective were comparable to the numbers stated by previous studies [22, 23]. Even though statistical significance was not reached, partners of ABR patients did more often strongly agree with the statement that the BR has improved their partners QoL. Depending on irradiation of the breast, type of implant and surgical technique [24] 10–45% of IBR patients develop a major capsular contracture (CC) which might be the most compromising factor of QoL [25, 26]. The autologous reconstructed breast has a natural, warm consistency and follows the natural aging process as well as weight changes. This results in a high level of acceptance and identification with the transplant, which has been reflected in previous studies reporting superior QoL in ABR patients compared to IBR [5, 6, 22]. Accordingly, as supported by our results, a central advantage of ABR is the softness of the breast(s) and how natural they feel to touch, which might be further affected in patients with CC. Furthermore, in selected patients with a thin skin envelope, the combination of an implant with lipofilling, mesh or acellular dermal matrix might be an option to improve breast softness in IBR [27, 28].

Especially in bilateral reconstruction, the more challenging and additionally symmetrical shaping of the new breasts with autologous tissue compared to the use of two identical implant-prosthesis might be the reason for the superiority of IBR in terms of partners evaluation of breast size, how the patient looks in clothes and with her able to wear clothes that is more fitted [29]. Both the lack of volume and an unsatisfactory breast shape are the underlying reasons for the oftentimes secondary procedures performed in ABR such as lipofilling [5, 29]. This finding is in line with a study published by Wallner et al. [30] investigating the perfect breast based on a large online survey showing that both men and women preferred an upper pole prominence as well as men preferring a breast cup size larger than B. Both characteristics of a rather artificially improved breast shape lacking the natural aging process which, at the same time, can be also disadvantageous in IBR in unilateral reconstruction [31].

Even though patient and partner’ satisfaction with breast and overall outcome were highly correlated, partners scored significantly higher. The reason might be the high demands women tend to set on their self and body [32]. Even though scores evaluating patient’s body acceptance and attractiveness were still high, they were rated lowest within all subscales in the category psychosocial well-being. This indicates that even though patients are confident in a social setting, feel emotionally stable and feminine in their clothes, body image and acceptance of the ‘new body’ present the major issue after breast reconstruction. Interestingly enough, the perceived psychosocial well-being of the patients was significantly overestimated by their partners which can be indicative of patients pretending to be more emotionally stable than they actually are. The offer of psycho-oncological support has been prevailed as part of the standard care in breast cancer. We suggest the inclusion of patient’s partners, to further sensitize them of the high psychological burden a breast cancer diagnosis goes along with and therefore stabilize patient’s private support system.

### Limitations

Limitations of the study include the cross-sectional design and the small sample size. Since questionnaires had to be completed in the home environment, due to corona pandemic restrictions, we cannot guarantee a setting in which partners were separated from the patients while answering the questionnaire. Moreover, we did not evaluate the psychological effects of a breast cancer diagnosis and associated treatment on the partner him/herself. This, including the relationship of patient and partner’s sexual well-being, should be investigated in future studies.

## Conclusion

In terms of partner satisfaction with the outcome both reconstructive procedures, ABR and IBR, achieve satisfactory results. ABR scored higher in the categories softness of breast and how natural the breast feels to touch whereas IBR was rated superior evaluating the breast size. Partners of ABR patients did estimate more often that the BR did improve the patient’s QoL. Partner and patient’s satisfaction with the breast, outcome as well as patient’s perceived and self-report psychosocial well-being were strongly associated with the partners scoring significantly higher. We recommend to include partners in the regular psycho-oncological support to further sensitize them of the high psychological burden of a breast cancer diagnosis and therefore stabilize patients private support system.

## Data Availability

The datasets generated and analyzed during the current study are not publicly available due to the privacy policy of the participating hospitals but are available from the corresponding author on reasonable request.
